# ANN-augmented adaptive droop/PI control for residential hybrid microgrids with IoT monitoring

**DOI:** 10.1038/s41598-026-46557-z

**Published:** 2026-04-10

**Authors:** Mohammed O. Bahabri, Sreerama Kumar Ramdas, Hussam A. Banawi

**Affiliations:** Department of Electrical and Computer Engineering, Faculty of Engineering, King Abdulaziz University, Jeddah, Saudi Arabia

**Keywords:** Microgrid, ANN, Adaptive Control, Droop Control, PI Control, IoT, ThingSpeak, Residential Microgrid, Jeddah, Hybrid Microgrid, Energy science and technology, Engineering

## Abstract

In alignment with Saudi Arabia’s Vision 2030 goals for energy diversification, sustainability, and increased renewable energy penetration, this paper presents an ANN-assisted adaptive droop/Proportional–Integral (PI) control strategy for residential hybrid microgrids integrating photovoltaic (PV) systems, optional micro-wind turbines, and battery energy storage. The proposed scheme is designed for a grid-tied residential microgrid with islanding capability and is modeled and simulated in MATLAB/Simulink. By dynamically tuning droop coefficients and PI gains in real time, the controller enhances system adaptability under fluctuating renewable generation and rapidly varying residential loads. Simulation results demonstrate that, compared with conventional fixed-gain drop/PI control, the proposed approach improves renewable energy utilization by approximately 15%, increases PV self-consumption by 20%, and reduces peak grid import by about 18%, while maintaining stable voltage and frequency regulation. Efficient energy shifting is achieved through coordinated battery operation, with charging during mid-day solar peaks and discharging during evening demand; battery power reaches up to 5 kW, and State of Charge (SoC) is maintained within 35–90% limits. An Internet of Things (IoT) monitoring layer implemented using ThingSpeak provides near real-time telemetry, with median communication latency below 500 ms and full command success rate. A detailed case study based on the climatic conditions and AC-dominated residential load profiles of Jeddah, Saudi Arabia confirms reduced PV curtailment, improved self-consumption, and decreased reliance on grid imports during peak demand periods. The results highlight the effectiveness of ANN-driven adaptive control combined with secure IoT monitoring in enhancing the performance and operational resilience of residential microgrids in challenging environments.

**Supplementary Information:**

The online version contains supplementary material available at 10.1038/s41598-026-46557-z.

## Introduction

Saudi Arabia is undergoing a comprehensive national transformation aimed at achieving economic diversification, sustainability, and technological advancement under Saudi Vision 2030. A central pillar of this vision is the modernization of the energy sector through large-scale integration of renewable energy resources, improved energy efficiency, and the deployment of intelligent power systems to ensure long-term reliability and resilience.

The global energy sector is undergoing a rapid transition toward low-carbon and decentralized power systems, driven by the increasing penetration of renewable energy sources (RESs) and the need for enhanced reliability and resilience. In this context, microgrids have emerged as a key enabling technology, providing localized energy systems capable of operating in both grid-connected and islanded modes while integrating distributed energy resources (DERs) such as photovoltaic (PV) systems, wind turbines, and battery energy storage systems (BESSs)^1,2^. Residential microgrids, in particular, play a crucial role in future smart grids by empowering end-users with energy autonomy, improved power quality, and reduced dependency on centralized generation^3^.

Despite their advantages, the operation of renewable-rich microgrids poses significant technical challenges. The inherent intermittency of solar and wind resources, combined with rapidly varying residential loads, can lead to voltage and frequency deviations, power-sharing inaccuracies, and reduced system stability^4,5^. These challenges become more pronounced in inverter-dominated microgrids, where the absence of inherent synchronous generator inertia increases the sensitivity of the system to disturbances. Although conventional droop and fixed-gain PI controllers are widely employed due to their simplicity and decentralized implementation, their performance may degrade under varying operating conditions and network uncertainties, particularly in low-voltage microgrids^6^.

To overcome these limitations, adaptive and intelligent control strategies have attracted growing attention in recent years. In particular, Artificial Neural Networks (ANNs) have demonstrated strong potential in enhancing microgrid control due to their ability to learn nonlinear relationships and adapt controller parameters online. Several studies have reported that ANN-based droop or PI tuning can significantly improve voltage regulation, frequency stability, and active/reactive power sharing compared to fixed-parameter controllers^7–9^. Early ANN-assisted droop control approaches focused on accurate power sharing in islanded microgrids^7^, while more recent works have explored hybrid ANN-based adaptive droop–PI strategies capable of handling fast renewable fluctuations and load transients^8,9^. Nevertheless, many existing ANN-based solutions rely on complex architectures or large training datasets, which may limit their real-time applicability in residential-scale microgrids.

Beyond primary control, hierarchical control architectures have been widely adopted to coordinate microgrid operation across multiple layers. The hierarchical framework—comprising primary, secondary, and tertiary control levels—enables fast local stability, voltage/frequency restoration, and higher-level energy management, respectively^10,11^. Comprehensive reviews have highlighted that integrating adaptive intelligence into hierarchical control layers can substantially enhance microgrid flexibility and robustness^5,12^. However, practical implementations that combine lightweight ANN-based adaptation with hierarchical control in residential microgrids remain relatively limited.

Research has demonstrated successful applications of ANNs in microgrids, from forecasting to direct adaptive tuning of inverter controls, including ANN-based droop coefficient adjustment and State-of-Charge (SoC) management^7,13^.

In parallel with advances in control techniques, the Internet of Things (IoT) has become a transformative technology for monitoring and managing distributed energy systems. IoT platforms enable real-time data acquisition, cloud-based visualization, and remote access to microgrid operational variables, thereby improving situational awareness and decision-making^14^. Concurrently, the integration of the Internet of Things (IoT) is revolutionizing microgrid management by enabling ubiquitous data acquisition, remote monitoring, and control. IoT platforms facilitate seamless communication between physical assets and cloud-based analytics, providing critical visibility and user-friendly interfaces for homeowners and operators^15,16^. Cloud-based services like ThingSpeak offer accessible solutions for data visualization and remote command execution, making advanced monitoring feasible for residential applications^17^. However, connecting critical energy infrastructure to the internet introduces significant cybersecurity risks, including data breaches and unauthorized control, mandating the integration of robust security frameworks from the design phase^18^.

The increasing connectivity of microgrids also introduces critical cybersecurity challenges, as IoT-enabled energy systems become exposed to cyber threats such as unauthorized access, data manipulation, and denial-of-service attacks^19,20^. International standards, particularly the IEC 62443 series, provide a structured framework for securing industrial automation and control systems and are increasingly recognized as relevant to microgrid and smart grid applications^21^. However, cybersecurity considerations are often overlooked in residential microgrid studies, or treated separately from control and monitoring design, despite their importance for reliable and trustworthy operation^22^.

Energy management under uncertainty frameworks have emerged to improve decision-making in hybrid microgrids in the presence of forecasting and modelling errors^23^. Recent research also explores IoT-enabled monitoring systems for enhanced parameter supervision and protective functionality in smart grids^24^.

Recent advancements in artificial intelligence (AI), particularly Artificial Neural Networks (ANNs), offer a promising avenue to address the limitations of conventional microgrid control. ANNs possess the capability to learn complex, non-linear relationships from data, enabling them to adapt control parameters in real-time to optimize performance under varying operating conditions. This adaptive capability can significantly enhance the dynamic response, stability, and power quality of microgrids, surpassing the performance of fixed-gain controllers^4,25^. Concurrently, the integration of Internet of Things (IoT) technologies is revolutionizing microgrid management by facilitating real-time data acquisition, remote monitoring, and control. IoT platforms enable seamless communication between microgrid components and external interfaces, providing operators and homeowners with critical insights and control capabilities via user-friendly dashboards^3,14^. Nevertheless, the deployment of IoT in critical energy infrastructure mandates rigorous attention to cybersecurity, ensuring data integrity, system availability, and protection against malicious attacks^14,16^.

Motivated by these gaps, this paper proposes an ANN-augmented adaptive droop/PI control framework integrated within a hierarchical microgrid architecture, complemented by secure IoT-based monitoring. A compact, data-efficient Multi-Layer Perceptron (MLP) is employed to adapt droop coefficients and PI gains online, enhancing voltage and frequency regulation, power sharing accuracy, and dynamic response under rapid renewable and load variations. The control framework is combined with a MATLAB/Simulink–ThingSpeak monitoring pipeline that provides real-time visualization while adhering to an IEC 62443-inspired cybersecurity checklist.

This paper proposes an innovative ANN-augmented adaptive droop/PI control strategy for residential hybrid microgrids, designed to address the aforementioned challenges within the context of Jeddah, Saudi Arabia. Our primary contributions include the introduction of a compact, data-light Multi-Layer Perceptron (MLP) that dynamically adjusts the parameters of conventional droop and PI controllers, significantly improving voltage and frequency regulation along with precise power-sharing under fast disturbances typical of the Jeddah environment, such as sudden load changes and rapid PV output variations. Furthermore, an ANN-assisted PI controller is developed to facilitate accurate setpoint restoration and effective State-of-Charge (SoC) balancing across the microgrids´ converters, thereby optimizing overall system operation and battery longevity. We also establish a comprehensive MATLAB/Simulink to ThingSpeak pipeline for real-time data telemetry and authenticated remote control via smartphone dashboards, with a detailed assessment of IoT communication latency and reliability, complemented by an IEC-62443-style security checklist to ensure robust and cyber-resilient operation. The proposed system is evaluated to quantify its effectiveness in maximizing the renewable fraction and self-consumption, while simultaneously minimizing curtailment, thereby demonstrating its superior performance compared to conventional baselines. Finally, the entire framework is validated through a detailed case study focused on Jeddah, Saudi Arabia, incorporating its specific climatic conditions, AC-dominated residential load profiles, and unique PV transients (e.g., dust/cloud events), providing practical insights into the systems´ performance in a real-world, challenging operational environment.

The remainder of this paper is structured as follows: Sect. 2 provides a review of relevant literature on ANN-based microgrid control and residential IoT integration. Section 3 details the system model and the proposed hierarchical control architecture. Section 4 elaborates on the ANN design, training methodology, and safety considerations. Section 5 describes the IoT/ThingSpeak integration and the implemented security protocols. Section 6 presents the Jeddah case study, including data sources, scenarios, and parameters. Section 7 discusses experimental results, including ablation studies and latency analysis. Finally, Sect. 8 concludes the paper and outlines future research directions.

## Related work

The rapid evolution of microgrid technologies necessitates continuous advancements in control strategies and integration methodologies. This section provides a concise overview of pertinent literature, focusing on the application of Artificial Neural Networks (ANNs) in microgrid control, the integration of Internet of Things (IoT) for energy management, and the critical aspects of cybersecurity in such interconnected systems. The aim is to contextualize the proposed research within the existing body of knowledge and highlight the unique contributions of this work.

Artificial Neural Networks have emerged as a powerful tool for addressing the complex and dynamic control challenges inherent in modern microgrids. Their ability to learn intricate, non-linear relationships from data makes them particularly suitable for adaptive control applications where system parameters or operating conditions are subject to significant variations. Early applications of ANNs in microgrids primarily focused on forecasting renewable energy generation and load demand, thereby facilitating optimal energy management and dispatch^28^. More recently, ANNs have been increasingly employed directly within the control loops to enhance stability, improve power quality, and optimize power sharing among distributed energy resources (DERs). For instance, several studies have explored ANN based adaptive droop control mechanisms. These approaches typically involve using ANNs to dynamically adjust the droop coefficients of inverters, thereby improving voltage and frequency regulation un- der transient conditions^8,9^. The advantage of such adaptive schemes lies in their capacity to overcome the limitations of fixed-gain controllers, which often struggle to maintain optimal performance across a wide range of operating points or during sudden disturbances. However, a common challenge identified in literature pertains to the computational complexity and data requirements for training and deploying sophisticated ANN models, especially for real-time applications in resource constrained microgrids^12^. Our work distinguishes itself by proposing a compact, data-light MLP architecture, specifically designed for efficient online adaptation, thereby minimizing computational overhead while maximizing control efficacy. Beyond droop control, ANNs have also been applied to secondary control layers for voltage and frequency restoration, as well as for State-of-Charge (SoC) management of battery energy storage systems (BESS)^3^. These applications leverage the ANN’s predictive capabilities to anticipate system deviations and generate appropriate corrective signals, leading to more precise and responsive control actions. The integration of ANNs into hierarchical control structures represents a significant step towards more intelligent and autonomous microgrid operation.

Recent advancements in photovoltaic (PV) system control seek to simplify architecture while improving performance under dynamic grid conditions. A significant contribution is the work by Jiang et al. (2024), who proposed a unified, single-layer Finite Control Set Model Predictive Control (FCS-MPC) strategy. This innovative framework integrates Maximum Power Point Tracking (MPPT) and grid current regulation into one predictive scheme, eliminating the need for separate modulation stages and cascaded loops. By optimizing both power extraction and grid synchronization through a single cost function, their method demonstrates superior dynamic response and resilience to grid disturbances compared to traditional decoupled control architectures. This approach represents a promising direction for more robust and efficient grid-tied solar inverters^31^.

The proliferation of Internet of Things (IoT) technologies has revolutionized the monitoring and control of energy systems, including residential microgrids. IoT platforms enable ubiquitous connectivity, facilitating real-time data acquisition from various sensors, smart meters, and DERs, and providing remote control capabilities. This connectivity allows for enhanced visibility into energy flows, optimized energy consumption patterns, and improved demand-side management^14^. Numerous research efforts have demonstrated the benefits of IoT integration in microgrids, ranging from smart home energy management systems to large-scale grid modernization initiatives. IoT-enabled systems can provide homeowners with detailed insights into their energy usage, enable automated control of appliances, and facilitate participation in demand response programs^14^. Cloud-based IoT platforms, such as ThingSpeak, offer convenient solutions for data visualization, analysis, and remote command execution, making them attractive for residential applications due to their ease of deployment and accessibility^16^. Despite the clear advantages, the integration of IoT in critical energy infrastructure introduces significant challenges, particularly concerning data security, privacy, and communication reliability. The distributed nature of IoT devices and their connectivity to the internet expose microgrids to various cyber threats, including data breaches, denial-of-service attacks, and unauthorized control^26^. While some studies acknowledge these security concerns, comprehensive frameworks that integrate robust cybersecurity measures with real-time performance assessment are still evolving. Our research directly addresses this gap by incorporating an IEC-62443-style security checklist and rigorously evaluating the impact of IoT communication latency on overall system performance, thereby contributing to the development of more secure and reliable IoT enabled microgrids. Demand-side management (DSM) in residential buildings has also been explored to optimize energy costs while maintaining comfort^29^.

The increasing interconnectedness of microgrids with the broader energy infrastructure and the internet has brought cybersecurity to the forefront of research and development. Microgrids, as critical infrastructure, are vulnerable to cyberattacks that can compromise their stability, reliability, and operational integrity. The consequences of such attacks can range from data manipulation and service disruption to physical damage and widespread blackouts^19^. International standards, such as the IEC 62443 series, provide a comprehensive framework for securing industrial automation and control systems (IACS), which are highly relevant to microgrid applications. These standards cover various aspects of cybersecurity, including risk assessment, security program development, system design, and secure component development^20^. While these standards offer valuable guidance, their application to residential microgrids, with their unique scale and resource constraints, requires careful consideration. Many existing residential microgrid implementations often overlook robust cybersecurity measures, prioritizing functionality and cost effectiveness over security^22^. Our work emphasizes the critical importance of cybersecurity by integrating an IEC-62443-inspired security framework into the IoT communication pipeline. This includes implementing measures such as API key management, Transport Layer Security (TLS) encryption, command whitelisting, rate-limiting, and the design of fail-safe mechanisms to ensure system resilience against cyber threats. By explicitly addressing these security considerations, our research aims to contribute to the development of more trustworthy and resilient residential microgrid systems, paving the way for their broader adoption and integration into the smart grid ecosystem.

As summarized in Table 1, prior studies have made important strides in adaptive control, monitoring, and cybersecurity for microgrids. However, most approaches address these challenges in isolation focusing either on ANN-based adaptation, IoT visualization, or basic security provisions. In contrast, the proposed work uniquely integrates a compact ANN architecture for real-time adaptive control with secure, IoT-enabled monitoring under an IEC-62443-inspired framework, all tailored for residential hybrid microgrids in dynamic environments such as Jeddah. This integrated approach offers a more robust and practical solution for enhancing microgrid autonomy, reliability, and resilience.

**Table 1:** Comparative work and method.

Representative works & methods	Representative works & methods	Key contributions/findings	Limitations/differences vs. this work
Adaptive droop control using ANN ANN & soft computing in secondary control & SoC management	Vigneysh & Kumarappan (2016)^7^ Encyclopedia MDPI entry (2024)	FFNN-based droop control optimizes the droop parameters to obtain accurate real/reactive power sharing without voltage and frequency swings The MPC-based microgrid control is supplemented with soft-computing methods such as fuzzy logic, ANN, and PSO, thus improving the resistance to nonlinearities and communication delays	Islanded focus; high cost of computation; no IoT/security features Prediction based method using large data sets; no droop/PI adaptation schemes
Microgrid monitoring IoT implementation	Benavides et al. (2023)	ThingSpeak-coordinated IoT sensors provide real-time and continuous photovoltaic and meteorological data, which can be used to make informed decisions regarding energy-management	Focus on monitoring/visualization only; no adaptive control and cybersecurity integration
Cybersecurity frameworks	Sustainability directory (2025)	The IEC 62,443 standard is a systematic set of security specifications of industrial control systems and smart grids, which covers the life-cycle security issues and the responsibilities of stakeholders	Industrial oriented focus; complicated implementation; not residential microgrids oriented
Current study (Bahabri et al. 2025)	Bahabri et al. (2026)—Compact MLP with IoT & IEC-62443-inspired security	Compact MLP tunes droop/PI gains online; integrates IoT dashboards with latency analysis; implements an IEC-62443- inspired security framework	N/A (This work)

## System model and control architecture

**Fig. 1:**
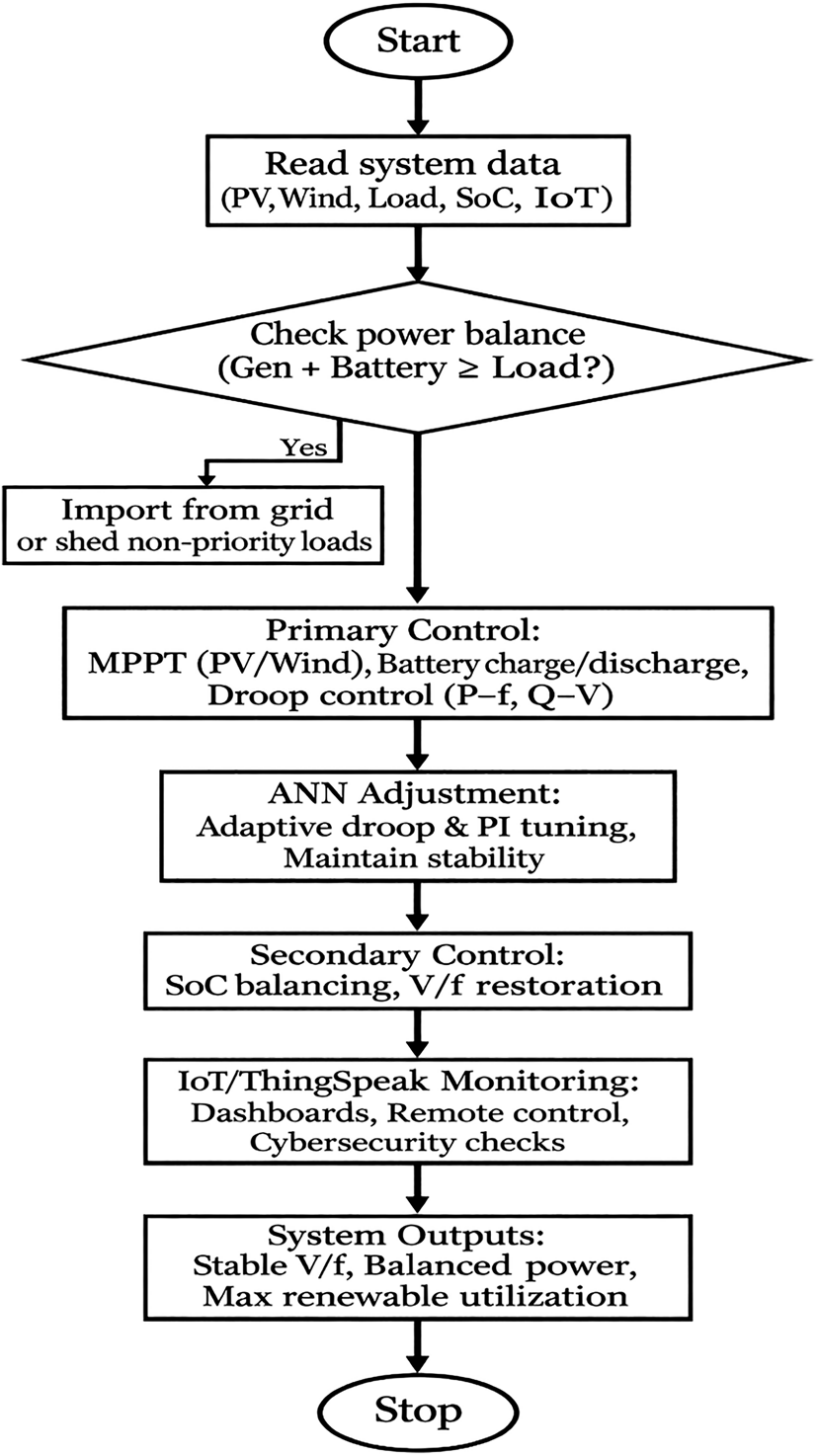
Flowchart of the proposed ANN-augmented Energy Management System (EMS).

This part discusses an integrated system model of the residential hybrid micro-grid and hierarchical control structure. The micro-grid is imagined as a one-home energy system, which is designed to work smoothly in both grid-connected and islanded system, and combines various distributed energy resources (DERs) to increase energy autonomy, reliability, and sustainability. The proposed residential micro-grid provides a hybrid AC/DC bus topology, which provides greater flexibility and efficiency in the incorporation of multiple DERs. The general functioning of the proposed artificial-neural-network-enhanced Energy Management System (ANN-EMS) of the residential hybrid micro-grid is demonstrated in Fig. 1, which shows the hierarchical control levels, ANN-based adaptive tuning, and IoT-based monitoring and security solutions.

The main elements of such a micro-grid are a rooftop photovoltaic (PV) array (1015 kW), an optional micro-wind turbine (13 kW), a battery energy storage system (BESS) (1020 kWh), AC loads (with a large air-conditioning load in Jeddah), and optional DC loads. The AC bus is connected to the main utility grid with a grid-tie inverter that handles power importation/exportation and transition between grid-connected and islanding. The hybrid AC/DC bus structure allows the direct connectivity of DC-based DERs and loads and, thus, minimizes conversion losses and enhances the overall system efficiency. Each of the DERs is fitted with power-electronic converters that have local controllers to maintain stability of operations and to interface well with the micro-grid buses^2,5^.

To aid the analysis and design of the control, the model of each micro-grid element is mathematically modeled, with emphasis on the power-electronic interfaces that control the power flow and interaction in the micro-grid. The power (P) of PV system, P_PV_ depends mainly on the solar irradiance, G, and ambient temperature, T. An over-simplified model can be written as:

1 $${P}_{PV}={P}_{PV,rated}\cdot \frac{G}{{G}_{ref}}\cdot [1+{k}_{T}(T-{T}_{ref})]$$

In which ${P}_{PV,rated}$, rated is the rated power at reference conditions (${G}_{ref}$, ${T}_{ref}$), and ${k}_{T}$ is the temperature coefficient of power. A DC-link voltage of the PV inverter is usually controlled by a maximum power point tracking (MPPT) algorithm to obtain maximum power. BESS dynamics are important to the stability of micro-grids and energy management. The State-of-charge (SoC) of the battery may be formulated as:

2 $$SoC\left(t\right)=SoC\left({t}_{0}\right)-\frac{1}{{C}_{batt}}{\int }_{{t}_{0}}^{t}\frac{{P}_{batt}\left(\tau \right)}{{\eta }_{batt}{V}_{batt}}d\tau$$

In which, ${C}_{batt}$ is battery capacity, ${P}_{batt}$ is battery power (positive discharge, negative charge), and ${\eta }_{batt}$ is the charging discharging efficiency, and ${V}_{batt}$ is the battery voltage. The present SoC and ${C}_{batt}$ capabilities of BESS are the limiting factors in terms of its power limits. The main interface of DERs in AC micro-grids is Voltage-Source Converters (VSCs). Droop control controls their behavior as a result of the connection between active/reactive power and frequency/voltage, respectively. In the case of an AC micro-grid, the standard P-f and Q–V droop curves can be expressed as:

3 $$\omega -{\omega }^{*}=-{m}_{p}\left(P-{P}^{*}\right)$$

4 $$V-{V}^{*}=-{n}_{q}\left(Q-{Q}^{*}\right)$$

In which the measured angular frequency and voltage magnitude are denoted by respectively, and the nominal values by respectively, the active and reactive power output is denoted by and respectively, the setpoints by the nominal angular frequency and voltage respectively, and the droop coefficients are denoted by and respectively. These coefficients are used to establish sensitivity of frequency to active power and voltage to reactive power respectively. Adaptive characteristic of the proposed control is in the dynamic control of the ANN of the values of the m_p_ and n_q_. In the case of DC micro- grids, the equivalent droop control relates the output current to the DC bus voltage:

5 $${V}_{dc}-{V}_{dc}^{*}=-{m}_{dc}\left({I}_{dc}-{I}_{dc}^{*}\right)$$

and where ${V}_{dc}$ and ${I}_{dc}$ are the DC bus voltage and current, ${V}_{dc}^{*}$ and ${I}_{dc}^{*}$ are their nominal values, and ${m}_{dc}$ is the DC droop coefficient. The general state of power balance in the micro-grid should be ensured. In the case of an AC micro-grid, the equations of the active and reactive power balance are:

6 $${P}_{grid}+\sum {P}_{DER}={P}_{load}+{P}_{loss}$$

7 $${Q}_{grid}+\sum {Q}_{DER}={Q}_{load}+{Q}_{loss}$$

where the active and reactive power exchanged with the main grid is denoted by ${P}_{grid}$ and ${Q}_{grid}$, the sums of the active and reactive power generated by all DERs (PV, wind, BESS) are denoted by sum $\sum {P}_{DER}$ and sum $\sum {Q}_{DER}$, active and reactive power loads are denoted by ${P}_{load}$ and ${Q}_{load}$, and losses (active and reactive) within the micro grid distribution lines and converters are denoted by ${P}_{loss}$ and ${Q}_{loss}$. The simulation environment is based on these mathematical models as well as the design of the ANN-augmented control algorithms. The dynamic characteristics of the proposed control directly affect the parameters of the proposed control, which is ${m}_{p}$, ${n}_{q}$, and the gain of the PI controllers, and hence, optimize the power flow and stability of the micro-grid in the case of dynamic conditions.

In this section, we also present detailed circuit- and control-level equations commonly used in the analysis and design of low-voltage residential hybrid microgrids. The presentation follows the synchronous $dq$ reference frame convention with $abc\to \alpha \beta \to dq$ Park transformations. Let $\theta$ denote the reference angle. The Park transform from stationary $\alpha \beta$ to synchronous $dq$ frame is:

8 $$\left[\begin{array}{l}{x}_{d}\\ {x}_{q}\end{array}\right]=\left[\begin{array}{ll}cos\theta & sin\theta \\ -sin\theta & cos\theta \end{array}\right]\left[\begin{array}{l}{x}_{\alpha }\\ {x}_{\beta }\end{array}\right], \dot{\theta }=\omega .$$

The instantaneous three-phase active and reactive powers in the dq frame (with peak-valued quantities

9 $$P=\frac{3}{2}\left({v}_{d}{i}_{d}+{v}_{q}{i}_{q}\right),$$

10 $$Q=\frac{3}{2}\left({v}_{q}{i}_{d}-{v}_{d}{i}_{q}\right).$$

Consider a VSC connected to the PCC through an $L$ filter with series resistance $R$ and inductance $L$. Let ${\boldsymbol{v}}=[{v}_{d} {v}_{q}{]}^{\mathrm{T}}$ be the converter output voltage command, ${{\boldsymbol{v}}}_{o}$ the PCC voltage, and ${\boldsymbol{i}}=[{i}_{d} {i}_{q}{]}^{\mathrm{T}}$ the inductor current. The continuous-time model is:

11 $$L {\dot{i}}_{d}={v}_{d}-{v}_{o,d}+\omega L {i}_{q}-R{i}_{d},$$

12 $$L {\dot{i}}_{q}={v}_{q}-{v}_{o,q}-\omega L {i}_{d}-R{i}_{q}.$$

A cascaded current controller (inner loop) with feedforward and decoupling is:

13 $${v}_{d}^{*}={v}_{o,d}-\omega L {i}_{q}+R{i}_{d}+{K}_{p,i}\left({i}_{d}^{\star }-{i}_{d}\right)+{K}_{i,i} \int \left({i}_{d}^{\star }-{i}_{d}\right)dt,$$

14 $${v}_{q}^{*}={v}_{o,q}+\omega L {i}_{d}+R{i}_{q}+{K}_{p,i}\left({i}_{q}^{\star }-{i}_{q}\right)+{K}_{i,i} \int \left({i}_{q}^{\star }-{i}_{q}\right)dt.$$

The outer (power/voltage) loop generates current references ${i}_{d}^{\star },{i}_{q}^{\star }$ according to power or voltage regulation objectives. For an LCL filter with converter-side inductance ${L}_{1}$, grid-side inductance ${L}_{2}$, damping resistance ${R}_{d}$ (in series with capacitor), and filter capacitance ${C}_{f}$, the undamped resonance frequency is:

15 $${\omega }_{res}=2\pi {f}_{res}=\sqrt{\frac{{L}_{1}+{L}_{2}}{{L}_{1}{L}_{2}{C}_{f}}}, \zeta \approx \frac{{R}_{d}}{2}\sqrt{\frac{{C}_{f}}{{L}_{1}+{L}_{2}}}.$$

Proper selection of ${R}_{d}$ (or active damping) is necessary to avoid oscillations near ${\omega }_{res}$. Let ${C}_{dc}$ be the DC-link capacitor, ${v}_{dc}$ its voltage, ${i}_{dc,in}$ the net current from sources (PV, battery via DC/DC), and ${i}_{dc,out}$ the VSC draw. Then:

16 $${C}_{dc}\frac{d{v}_{dc}}{dt}={i}_{dc,in}-{i}_{dc,out}, {p}_{dc}={v}_{dc}{i}_{dc,out}\approx \frac{\frac{3}{2}\left({v}_{d}{i}_{d}+{v}_{q}{i}_{q}\right)}{{\eta }_{inv}}.$$

A common SRF-PLL locks the $q$-axis PCC voltage to zero. With PI compensator ${G}_{pll}\left(s\right)$

17 $${\omega }_{pll}\left(s\right)={\omega }^{*}+{G}_{pll}\left(s\right){e}_{pll}\left(s\right),$$

18 $$\dot{\theta }={\omega }_{pll}.$$

Enhancing classic droop with virtual synchronous machine (VSM) dynamics yields:

19 $$M\dot{\omega }={P}^{\star }-P-D\left(\omega -{\omega }^{*}\right)-{m}_{p}\left(P-{P}^{\star }\right),$$

20 $${T}_{v}\dot{V}={V}^{\star }-V-{n}_{q}\left(Q-{Q}^{\star }\right)-{D}_{v}\left(V-{V}^{*}\right),$$

where $M$ is virtual inertia, $D$ is damping, and ${T}_{v},{D}_{v}$ are voltage-loop constants. Setting $M = 0,D = 0$ recovers standard droop (7)–(8). For a Thevenin source $E\angle \delta$ feeding a bus $V$ through impedance $Z=R+jX$, the approximate steady-state power relations are:

21 $$P\approx \frac{EV}{\left|Z\right|}\mathrm{sin}\left(\delta -\phi \right)-\frac{{V}^{2}}{\left|Z\right|}\mathrm{sin}\left(-\phi \right),$$

22 $$Q\approx \frac{EV}{|Z|}\mathrm{cos}(\delta -\phi )-\frac{{V}^{2}}{|Z|}\mathrm{cos}(-\phi ), \phi =\mathrm{arctan}\left(\frac{X}{R}\right).$$

When $\frac{R}{X}$ is non-negligible (typical in LV networks), $P$ and $Q$ both couple to $V$ and $\delta$, affecting droop tuning and power sharing. A first-order Thevenin model for the battery yields terminal voltage:

23 $${v}_{t}={E}_{oc}\left(SoC\right)-{R}_{0}i-{v}_{1},$$

24 $${\dot{v}}_{1}=-\frac{1}{{R}_{1}{C}_{1}}{v}_{1}+\frac{{R}_{1}}{{C}_{1}}i,$$

25 $$\dot{S}oC=-\frac{\eta i}{{C}_{nom}},$$

with open-circuit voltage ${E}_{oc}\left(\cdot \right)$, ohmic resistance ${R}_{0}$, RC branch $\left({R}_{1},{C}_{1}\right)$, and coulombic efficiency $\eta$. The PV cell I–V relation with series ${R}_{s}$ and shunt ${R}_{sh}$ resistances is:

26 $$i={I}_{ph}-{I}_{0} \left[exp \left(\frac{v+i{R}_{s}}{n{V}_{T}}\right)-1\right]-\frac{v+i{R}_{s}}{{R}_{sh}}, {V}_{T}=\frac{kT}{q}{N}_{s}.$$

The maximum power point satisfies $\frac{dP}{dv}=0$, i.e.,

27 $$\frac{dP}{dv}=i+v \frac{di}{dv}=0 \Rightarrow \frac{di}{dv}=-\frac{i}{v},$$

which underpins the incremental conductance MPPT law. To remove steady-state droop errors, integral secondary control acts on frequency and voltage references:

28 $$\dot{\Delta }{\omega }^{sec}=-{K}_{i,\omega } \left(\omega -{\omega }^{*}\right), {\omega }^{ref}={\omega }^{*}+\Delta {\omega }^{sec},$$

29 $$\dot{\Delta }{V}^{sec}=-{K}_{i,V} (V-{V}^{*}), {V}^{ref}={V}^{*}+\Delta {V}^{sec}.$$

Over a horizon $k=0,\dots ,H$, the energy cost and degradation proxy can be minimized:

30 $$\mathrm{min}\sum_{k}({c}^{imp}{P}_{k}^{imp}-{c}^{exp}{P}_{k}^{exp})\Delta t+{c}^{deg} \sum_{k}|{P}_{k}^{batt}|\Delta t$$

subject to:

31 $$So{C}_{k+1}=So{C}_{k}+\frac{{\eta }_{ch}{P}_{k}^{batt+}-\frac{{P}_{k}^{batt-}}{{\eta }_{dis}}}{{E}_{nom}} \Delta t,$$

32 $$0\le So{C}_{k}\le 1, 0\le {P}_{k}^{batt+}\le {P}_{max}^{batt}, 0\le {P}_{k}^{batt-}\le {P}_{max}^{batt},$$

33 $${P}_{k}^{imp},{P}_{k}^{exp}\ge 0, {P}_{k}^{imp} {P}_{k}^{ex\mathrm{p}}=0,$$

34 $${\mathrm{P}}_{\mathrm{k}}^{\mathrm{load}}={\mathrm{P}}_{\mathrm{k}}^{\mathrm{PV}}+{\mathrm{P}}_{\mathrm{k}}^{\mathrm{wind}}+{\mathrm{P}}_{\mathrm{k}}^{\mathrm{batt}-}-{\mathrm{P}}_{\mathrm{k}}^{\mathrm{batt}+}+{\mathrm{P}}_{\mathrm{k}}^{\mathrm{imp}}-{\mathrm{P}}_{\mathrm{k}}^{\mathrm{exp}}-{\mathrm{P}}_{\mathrm{k}}^{\mathrm{curt}},$$

35 $$0\le {\mathrm{P}}_{\mathrm{k}}^{\mathrm{curt}}\le {\mathrm{P}}_{\mathrm{k}}^{\mathrm{PV}}.$$

The above set of models provides a comprehensive analytical foundation for control design, stability assessment, and scheduling in residential hybrid microgrids, enabling reproducible tuning and rigorous performance verification^3,5^.

**Fig. 2:**
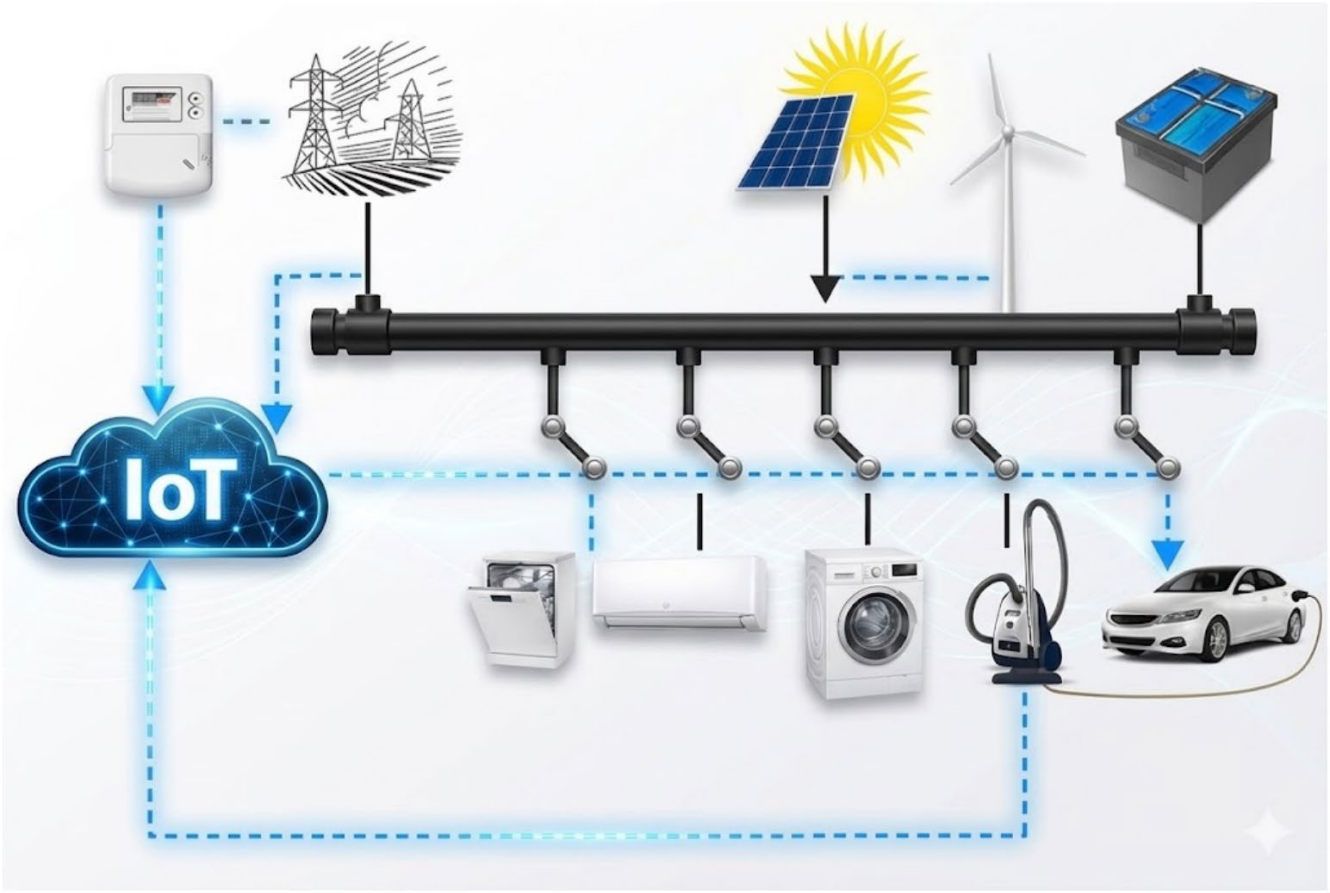
The system level diagram of the residential hybrid microgrid with IoT based monitoring and control.

The general design of the proposed residential hybrid micro-grid system is demonstrated in Fig. 2. The micro-grid incorporates solar PV mounted on the rooftop, optional micro-wind turbine and battery energy storage system (BESS), which is connected to local AC and optional DC loads via smart inverters. A grid tie in enables a two way exchange of power with the main utility. The local Energy Management System (EMS) is involved in control and coordination activities, and the IoT framework is used to conduct real-time monitoring, remote visualization, and secure communication to the cloud based on services like ThingSpeak. The architecture allows flexible and autonomous operation with greater visibility and resiliency.

The architecture includes PV, wind, battery storage, grid tie, and diverse AC loads managed through an EMS with cloud-based visibility.

## Proposed artificial neural networks: design and training aspects

This section details the design, training methodology, and the safety considerations for the proposed Artificial Neural Networks (ANNs) employed in the primary and secondary control layers of the residential hybrid microgrid. The objective is to develop compact, efficient, and robust ANNs that can adaptively tune controller parameters in real-time, thereby enhancing system performance without imposing excessive computational burden.

For the primary control layer, a Multi-Layer Perceptron (MLP) is utilized to adaptively adjust the droop coefficients (m_p_, n_q_) and/or the gains of the Proportional-Integral (PI) controllers. The design philosophy prioritizes simplicity and computational efficiency to ensure real-time implement ability on typical microgrid control hardware. The MLP architecture consists of an input layer, one or two hidden layers, and an output layer. The selection of input features for the MLP is crucial for its ability to accurately infer the optimal controller parameters. The in- puts are chosen to provide a comprehensive snapshot of the microgrid’s instantaneous operating state and dynamic deviations, including voltage deviation (∆V), frequency deviation (∆ f), active power deviation (∆P), reactive power deviation (∆Q), battery State-of-Charge (SoC), ambient temperature, PV irradiance, and load change rate. These inputs are normalized to a specific range (e.g., [0, 1] or [− 1, 1]) to ensure consistent scaling and improve the training process of the ANN. The outputs of the MLP are the adaptive scaling factors or direct adjustments to the droop coefficients and PI controller gains. For instance, the MLP might output a multiplier for m_p_ and n_q_, or directly output the proportional (K_p_) and integral (K_i_) gains for the PI controllers. Crucially, hard bounds are imposed on these outputs to ensure that the adjusted parameters remain within a safe and stable operating range, preventing any unstable control actions that could compromise microgrid stability. This bounding mechanism is a critical safety feature, especially in a real-time control environment. The number of hidden layers and neurons per layer is kept minimal (e.g., one hidden layer with 10–20 neurons) to reduce computational complexity. Sigmoid or ReLU activation functions are typically employed in the hidden layers due to their computational efficiency and effectiveness in capturing non-linear relationships.

The Multi-Layer Perceptron (MLP) employed for adaptive control can be mathematically described by a series of weighted sums and activation functions. For a single hidden layer MLP, the output of a neuron in the hidden layer, h j, is given by:

36 $${\mathrm{h}}_{\mathrm{j}}=\mathrm{f}\left(\sum_{\mathrm{i}=1}^{{\mathrm{N}}_{\mathrm{in}}}{\mathrm{w}}_{\mathrm{ji}}^{\left(1\right)}{\mathrm{x}}_{\mathrm{i}}+{\mathrm{b}}_{\mathrm{j}}^{\left(1\right)}\right)$$

where ${\mathrm{x}}_{\mathrm{i}}$ are the inputs from the input layer, ${\mathrm{w}}_{\mathrm{ji}}^{\left(1\right)}$ are the weights connecting input neuron $\mathrm{i}$ to hidden neuron $\mathrm{j}$, ${\mathrm{b}}_{\mathrm{j}}^{\left(1\right)}$ is the bias for hidden neuron $\mathrm{j}$, and $\mathrm{f}\left(\cdot \right)$ is the activation function (e.g., sigmoid or ReLU).

The output of the MLP, ${\mathrm{y}}_{\mathrm{k}}$, which represents the adjusted controller parameters (e.g., ${\mathrm{m}}_{\mathrm{p}}$, ${\mathrm{n}}_{\mathrm{q}}$, ${\mathrm{K}}_{\mathrm{p}}$, ${\mathrm{K}}_{\mathrm{i}}$), is then calculated from the hidden layer outputs:

37 $${\mathrm{y}}_{\mathrm{k}}=\mathrm{g}\left(\sum_{\mathrm{j}=1}^{{\mathrm{N}}_{\mathrm{hidden}}}{\mathrm{w}}_{\mathrm{kj}}^{\left(2\right)}{\mathrm{h}}_{\mathrm{j}}+{\mathrm{b}}_{\mathrm{k}}^{\left(2\right)}\right)$$

where ${\mathrm{N}}_{\mathrm{hidden}}$ is the number of neurons in the hidden layer, ${\mathrm{w}}_{\mathrm{kj}}^{\left(2\right)}$ are the weights connecting hidden neuron $\mathrm{j}$ to output neuron $\mathrm{k}$, ${\mathrm{b}}_{\mathrm{k}}^{\left(2\right)}$ is the bias for output neuron $\mathrm{k}$, and $\mathrm{g}\left(\cdot \right)$ is the output activation function (typically linear for regression tasks).

The training of the primary control ANN is performed offline using a comprehensive dataset generated from extensive MATLAB/Simulink simulations. This approach allows for thorough exploration of various operating conditions and disturbance scenarios, ensuring the ANN learns robust and generalizable control policies. The training dataset is generated by simulating the microgrid under a wide range of conditions, including diverse load profiles, renewable generation variability (e.g., rapid ramps due to dust storms and cloud transients), grid disturbances (e.g., voltage sags/swells, frequency deviations, islanding/reconnection events), and component faults. For each simulation run, the input features (e.g., ∆V, ∆f, SoC) and the corresponding optimal controller parameters (determined by an offline optimization algorithm or expert system) are recorded, creating a supervised learning dataset. The collected data is used to train the MLP using standard backpropagation algorithms, such as Adam or Levenberg–Marquardt. The training objective is to minimize the error between the ANN s predicted controller parameters and the optimal parameters from the simulation data. Techniques like early stopping and cross-validation are employed to prevent overfitting and ensure the model generalizes well to unseen data. During training, the ANN learns to map input states to optimal controller parameters by minimizing a loss function, typically the Mean Squared Error (MSE), between the ANN s predicted outputs and the target optimal parameters from the simulation data:

38 $$\mathrm{L}=\frac{1}{{\mathrm{N}}_{\mathrm{samples}}}\sum_{\mathrm{s}=1}^{{\mathrm{N}}_{\mathrm{samples}}}\sum_{\mathrm{k}=1}^{{\mathrm{N}}_{\mathrm{out}}}({\mathrm{y}}_{\mathrm{k},\mathrm{s}}^{\mathrm{pred}}-{\mathrm{y}}_{\mathrm{k},\mathrm{s}}^{\mathrm{target}}{)}^{2}$$

where ${\mathrm{N}}_{\mathrm{samples}}$ is the number of training samples, ${\mathrm{N}}_{\mathrm{out}}$ is the number of output parameters, ${\mathrm{y}}_{\mathrm{k},\mathrm{s}}^{\mathrm{pred}}$ is the ANN s predicted value for output $\mathrm{k}$ of sample $\mathrm{s}$, and ${\mathrm{y}}_{\mathrm{k},\mathrm{s}}^{\mathrm{target}}$ is the corresponding target optimal value. The weights and biases of the ANN are updated using an optimization algorithm, such as Adam, which iteratively adjusts the parameters to minimize $\mathrm{L}$

39 $${\theta }_{t+1}={\theta }_{t}-\alpha \cdot \frac{{\widehat{m}}_{t}}{\sqrt{{\widehat{v}}_{t}}+\epsilon }$$

Where θ represents the ANN parameters (weights and biases), α is the learning rate, ${\widehat{\mathrm{m}}}_{\mathrm{t}}$ and ${\widehat{\mathrm{v}}}_{\mathrm{t}}$ are the bias-corrected first and second moment estimates of the gradients, and ε is a small constant to prevent division by zero. This iterative optimization process allows the ANN to learn the complex relationships required for adaptive control. While the primary training is offline, a brief on-site fine-tuning mechanism can be incorporated, involving collecting a small amount of real-time operational data from the deployed microgrid to slightly adjust the pre-trained ANN weights. This fine-tuning helps to account for any discrepancies between the simulation model and the real-world system, further enhancing the ANN s performance and adaptability to site-specific characteristics. This process is designed to be quick and non-disruptive, potentially occurring during periods of low system stress.

For the secondary control layer, an ANN is employed to assist the PI controllers in setpoint restoration and State-of-Charge (SoC) balancing. This ANN can be a simpler MLP or even a Radial Basis Function (RBF) network, given the slower dynamics of the secondary control. Inputs to this ANN typically include system-wide voltage and frequency deviations, aggregate active and reactive power imbalances, and the SoC of all connected batteries. The outputs are corrective signals that adjust the reference power setpoints for the primary controllers or directly influence the integral terms of the secondary PI controllers. The goal is to drive the microgrid voltage and frequency back to nominal values and ensure equitable SoC distribution among batteries. The training for SoC balancing focuses on learning optimal charging and discharging strategies for the BESS based on factors like forecasted load, renewable generation, and battery health. The ANN aims to minimize battery degradation (e.g., by avoiding deep discharges or overcharging) while ensuring sufficient energy reserves for reliability. This can involve multi-objective optimization during training to balance energy management goals with battery longevity.

The ANN updates the controller parameters once per control cycle, synchronized with the secondary control layer, with an update interval of 50 ms. This update rate is intentionally selected to be slower than the primary control loop, ensuring stable real-time adaptation without parameter chattering or interference with fast inner-loop dynamics.

This part outlines the design and training approach of the Artificial Neural Network (ANN) that will be adopted in the proposed adaptive control strategy. The ANN is a key element to the real-time parameter adjustment, thus, allowing the Energy Management System (EMS) of the microgrid to react well to the new dynamics of the operating conditions, as well as optimize the movement of energy.

### ANN architecture

**Fig. 3:**
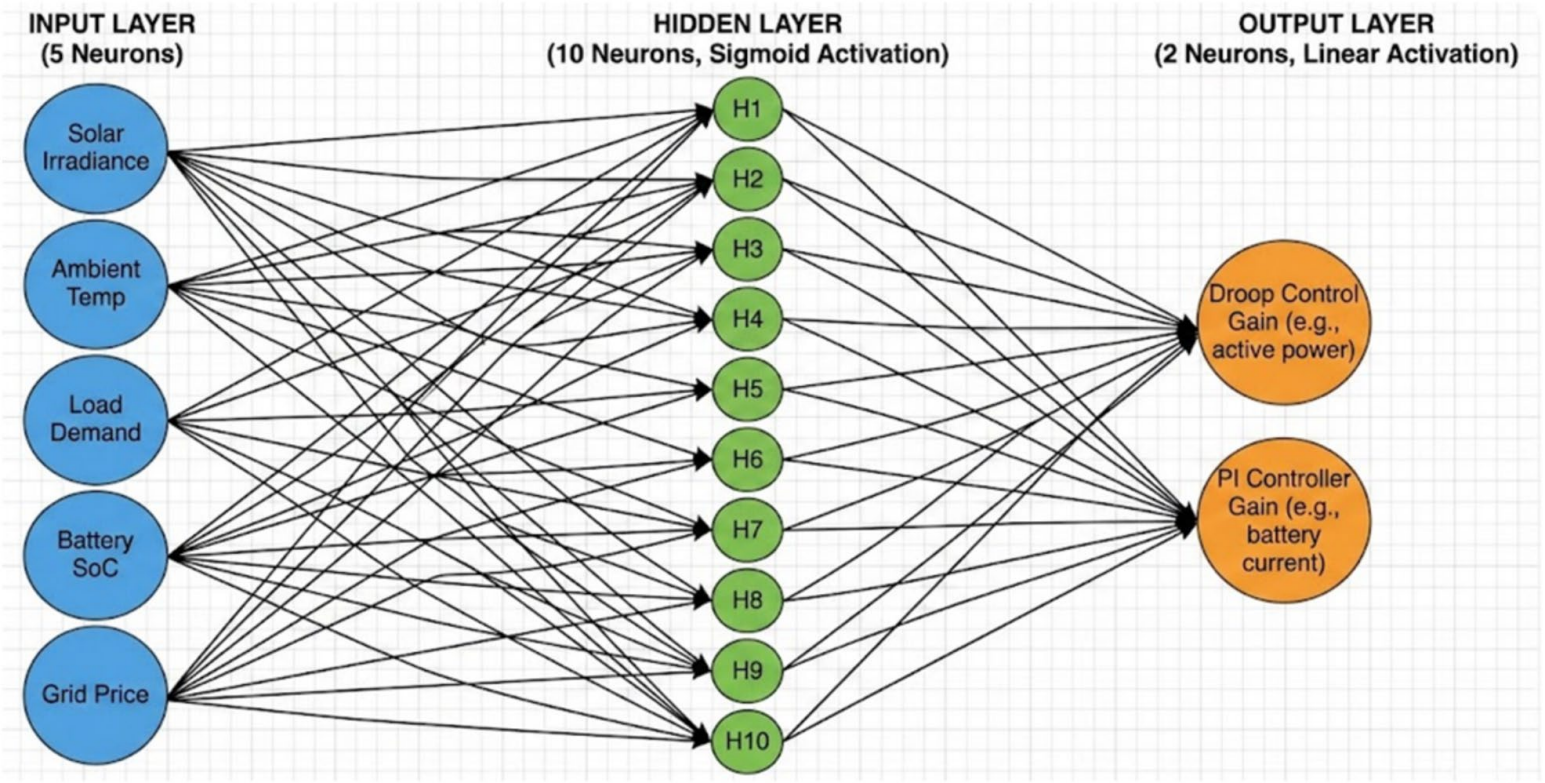
Artificial Neural Network (ANN) Architecture.

The adaptive control strategy consists of a single hidden layer Feedforward Neural Network (FFNN), which was chosen due to its trade-off between the complexity of the model and the computational cost. The architecture is described in Fig. 3 and it is structured into three layers.

Input Layer: It has five neurons each associated with a major parameter of operation: solar irradiance, ambient temperature, load demand, battery State-of-Charge (SoC), and grid price signal.

Hidden Layer: It contains ten neurons that make use of a sigmoid activation function. It is a layer specifically meant to draw out non-linear relationships that are complex in the input parameters and to process them so that they can be used to produce control outputs at a later stage.

Output Layer: Comprised of two neurons that produce the adaptive gains to droop control (e.g. the droop coefficient relating to active power sharing) and the Proportional-Integral (PI) controller (e.g. the proportional gain that controls battery current ). The output layer uses a linear activation function to ensure a smooth scale of values of gain that are adjustable.

The number of neurons in the hidden layer is optimized, which is essential for preventing over-fitting of the system and at the same time, providing enough learning capacity to the system.

### Training and dataset

The ANN is trained with a large scale dataset based on the extensive simulations of the residential microgrid under a wide range of operating conditions including changes in solar irradiance profiles, load demands, temperature variations, and grid price signals. The data set consists of around 10,000 data points, with each data point being a particular microgrid state and optimal control parameter values that were determined using an offline optimisation tool (e.g., Model Predictive Control or dynamic programming).

1. Data Preprocessing: Both input and output values were rescaled to the range [0, 1] to increase the stability of training and the speed of convergence, thus avoiding the situation when one input feature dominates the learning process because of its value.
2. Training Algorithm: The Levenberg–Marquardt back-propagation algorithm is used which is known to converge quickly with the network of moderate size. The data is divided into 70 percent training, 15 percent validation and 15 percent test.
3. Performance Metrics: The mean squared error (MSE) is the main performance measure used in the training process. Training was stopped once the error of validation stopped decreasing over a specified number of epochs which reduced over-fitting.

The trained ANN is then incorporated into the EMS of the microgrid providing real-time adaptive control parameters depending on the current operational state. This method allows the system to maintain an optimum performance despite unanticipated disturbances or changes in system dynamics, thus it does not require re-optimisation.

### MLP-MPPT performance evaluation

In order to increase the total system efficiency, a separate Multilayer Perceptron (MLP) is used as a Maximum Power Point Tracking (MPPT) algorithm of the PV system. This network is designed to replace traditional algorithms, including Perturb and Observe (P&O) and Incremental Conductance (IncCond), and the goal of this is to produce better energy extraction when the irradiance varied. Table 2 compares the performance of the proposed MLP-MPPT and traditional approaches.

**Table 2:** Performance of MPPT algorithms comparison.

Metric	MLP-MPPT	P&O	IncCond
Average Tracking Efficiency (%)	98.8	94.5	96.2
Energy Gain (kWh/24 h)	12.1	10.2	10.8
Response Time (s)	0.2–0.4	0.8–1.5	0.5–1.0
Power Ripple (%)	0.8	3.5	2.0

As shown in Table 2, the MLP-MPPT consistently outperforms both P&O and IncCond algorithms across all evaluated metrics. The average tracking efficiency of the MLP-MPPT reaches 98.8%, demonstrating its superior ability to accurately track the maximum power point even under rapid solar irradiance fluctuations, thereby directly contributing to the additional energy gain mentioned in the abstract.

A critical aspect of deploying ANNs in real-time control systems is ensuring safety and stability. The primary concern is to prevent the ANN from generating control signals that could lead to system instability or damage. To this end, hard output bounds are applied to the ANN outputs, derived from the physical limitations of the power electronic converters and the stability margins of the microgrid. Any output from the ANN that falls outside these bounds is clipped to the nearest valid limit. The ANNs are trained on data where the target outputs (optimal controller parameters) are known to maintain system stability and performance, ensuring that the ANN learns to operate within safe boundaries. Extensive testing under extreme operating conditions, including fault scenarios and high-stress transients, is performed during the simulation phase to validate the ANN’s robustness and its ability to maintain stability even when faced with unexpected inputs. Finally, the overall control system includes fail-safe mechanisms. In the un- likely event of an ANN malfunction or the generation of an unsafe control signal, the system can revert to a pre-defined, stable conventional control mode or initiate a controlled shutdown of affected DERs. This ensures that human intervention or system damage is prevented. It can be observed that the proposed ANN-augmented control strategy provides a reliable and high-performance solution for residential hybrid microgrids^12,27^. This approach ensures that the benefits of AI-driven adaptability are realized without compromising the critical stability and safety requirements of power systems.

## IoT/ThingSpeak integration and security

**Fig. 4:**
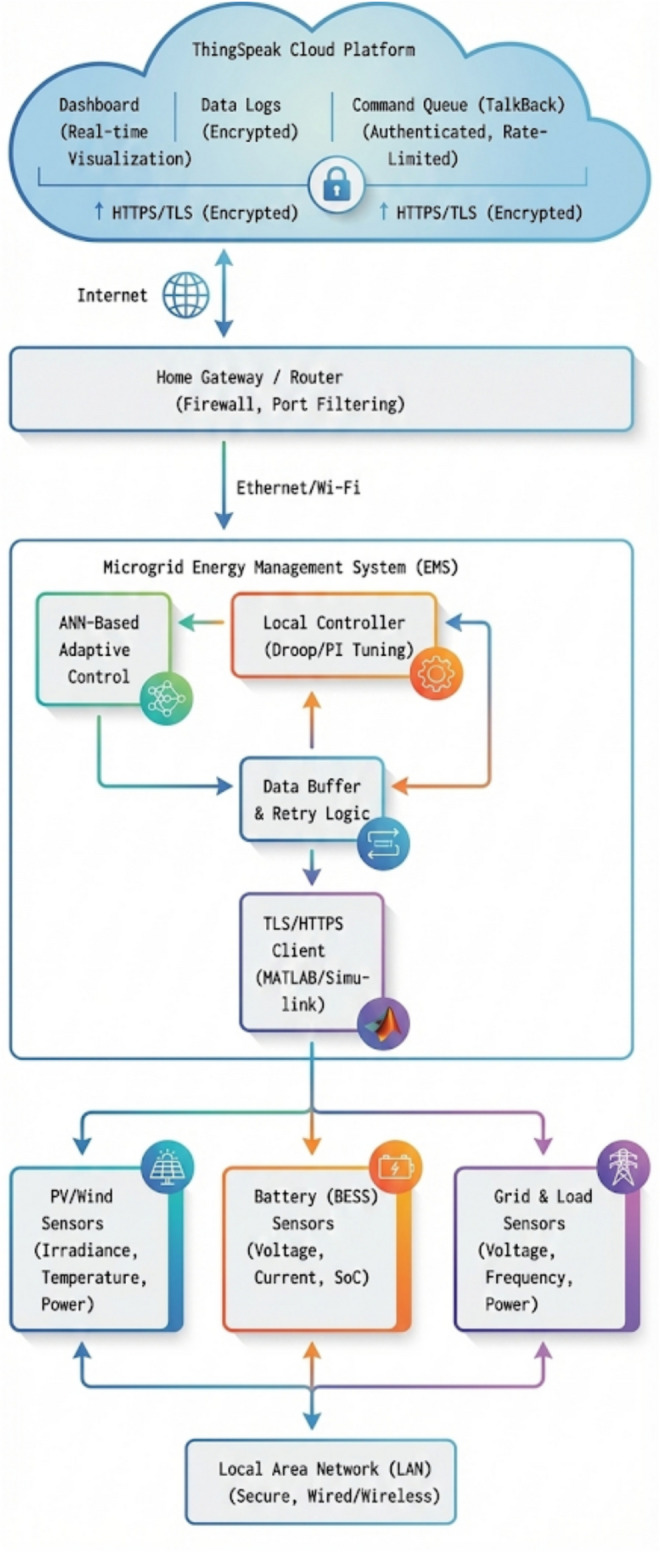
IoT Data Flow and Security Framework for the Residential Hybrid Microgrid.

The deployment of an Internet-of-Things (IoT) framework to monitor and remotely control a residential hybrid microgrid in real-time and integrate it with the ThingSpeak platform and the strong security controls to protect the integrity of data and system availability and resistance to cyber-threats are discussed in this Fig. 4 represents the overall data flow and security architecture of the IoT, showing the communication routes, the encryption points, and the fail-safe solutions connecting local sensors, the energy management system (EMS) and the cloud platform.

The IoT integration is designed in such a way that it provides full observability and controllability of the microgrid system. This ensures a smooth data flow between the MATLAB/Simulink simulation environment and the ThingSpeak cloud platform and then to user-friendly dashboards presented on smart phones or web browsers. In MATLAB/Simulink, the operational parameters of the microgrid that are identified as critical to real-time monitoring are active and reactive power flows (P/Q) at each node (PV, wind, battery, grid, load), battery State-of-Charge (SoC), microgrid voltage (V) and frequency (f), the status of each individual DER (e.g., PV output, wind speed) and alarm indicators (e.g., over-current, undervoltage, islanding event). These measurements are periodically sampled (e.g. every 1–5 s) to enable near real-time telemetry. MATLAB/Simulink uses its in-built ThingSpeak integration functionality or custom scripts to post the obtained telemetry to specified ThingSpeak channels, and communication is intermediated by the ThingSpeak REST API, mostly by means of HTTP POST requests. This will ensure that the operational information is sent safely and stored in the cloud. ThingSpeak has customizable visualisation and widgets that can be used to create user-friendly dashboards, which can be viewed online or through a special smartphone app, and thus allows homeowners and operators to view the key performance indicators (KPIs) of the microgrid in real time.

In addition to passive monitoring, the IoT platform allows authenticated remote control, whereby users can send command signals (e.g., change battery charging/discharging setpoints, switch between grid-connected and islanded modes, or activate/deactivate non-critical loads) to the MATLAB/Simulink platform through ThingSpeak. These commands are generally run in authenticated HTTP GET requests to the ThingSpeak API of the TalkBack or similar command queues, then read and processed by the Simulink model, and a rate-limit is placed on the number of command requests to discourage swamped command requests. One critical design issue is how the microgrid will act in case of loss of cloud-communication (e.g. loss of internet). The control system also includes a strong fail-safe system in which in case of a communication failure with ThingSpeak, the microgrid automatically switches to a local autonomous mode, thus ensuring that critical stability and power supply to essential loads are maintained and non-critical remote commands are temporarily disabled until communication is reestablished.

The systems are to be equipped with strong cybersecurity to prevent unauthorised access, manipulation of data, and disruption of the work. The IoT integration has a detailed security checklist that has been adopted in accordance with guidelines that are based on the IEC 62443 series of standards on industrial control systems. This includes the use of scope-limited and frequently rotated API keys to authenticate, and ensures that all data between MATLAB/Simulink and ThingSpeak are encrypted with TLS (HTTPS), preventing data being sent and received via the network. Remote command capabilities are restricted to a set of well-defined, non-critical commands through command whitelists, and rate-limited to prevent denial-of-service attacks or other fast, destabilising control-space changes^19,26^. The ThingSpeak system is set to send notifications when anomalous data patterns or unsuccessful attempts to execute commands are detected so that potential cyber incidents can be identified in time. The local microgrid controllers include watchdog timers, which undergo a safe fallback to local autonomous control when expected communications in the IoT platform have not been received in time, preventing the microgrid from entering an uncontrolled state (through communication loss or cyber-attack). Each and every data publication, command request, and security-related event is recorded on the local microgrid controller and the ThingSpeak platform, providing audit trails that can be used to analyze the post-incident analysis and verify compliance. Lastly, IoT integration custom scripts and interfaces are developed following the principles of secure coding, which reduces the likelihood of vulnerabilities in the form of injection vulnerability or insecure direct object references^20,22^. The IoT framework provides the residential hybrid microgrid with a chance to enjoy the benefits of remote monitoring and control without jeopardizing its cyber-resilience to a high level, thus supporting its safe and stable functioning in a networked environment by incorporating all these multilayered security layers.

### Communication reliability and security under grid faults

In addition to the implemented IEC 62443-inspired security measures, the system is designed to maintain communication integrity during grid disturbances. In the event of a grid fault or islanding transition, the IoT communication link remains secured via TLS 1.2/1.3 encryption, ensuring continued data confidentiality and integrity. To mitigate packet loss during transient faults, the MATLAB/Simulink client implements a buffering and retry mechanism with exponential backoff. Furthermore, the local EMS is programmed to prioritize control stability over cloud connectivity; if communication latency exceeds a predefined threshold (e.g., 2 s), the system autonomously switches to a pre-configured fail-safe mode, maintaining operational security without relying on cloud commands. This layered approach ensures both cyber-resilience and operational reliability under adverse grid conditions.

## Case study: data, scenarios, and parameters

To evaluate the effectiveness of the proposed ANN-enhanced adaptive control approach and IoT integration, a case study with the focus on a residential hybrid microgrid in Jeddah, Saudi Arabia, is performed. This section outlines the particular features of Jeddah that underlie the case study, the sources of data used, the simulation conditions, and the most important parameters of microgrid elements.

A large urban center with Red Sea coasts, Jeddah is a unique and problematic location to implement microgrids, due to its specific climatic and socioeconomic characteristics. The climate of the city is that of a hot arid coastal climate, where summer temperatures are regularly above 40 0 C. As a result, cooling of homes through air conditioners prevails, and peak loads are produced during summer afternoons and evenings. AC cycling has a significant effect on the load profiles and produces fast and significant load variations. In addition, the arid environment and geographical position expose the region to dust storms and cloud transients that may suddenly reduce the solar irradiance, and hence have a direct effect to PV output. The rooftop photovoltaic (PV) is the major renewable resource that is taken into account due to the high level of solar insolation. Although micro-wind resources in urban Jeddah are usually low- and medium-scale in nature, a non-compulsory micro-wind turbine is added to analyze the diversity of resources and its input at the time when the solar production is minimal or when particular wind events occur. Thermal flexibility, e.g. using heat-pump water heaters, pre-cooling plans, etc., is considered, with its ability to redistribute thermal loads and aid in demand-side management, but the major emphasis is on electrical load management. The residential micro grid is simulated as a grid tied system, which is indicative of the common connection of homes to the utility feeder. Scenarios include normal grid-connected operation and deliberate/accidental islanding events, and reconnection. Without publicly available detailed local tariffs or net-metering regulations, the transparent tariff situations (e.g., a flat rate, time-of-use, or peak pricing) are outlined to evaluate the economic consequences of grid interaction and energy-management policies.

Realistic simulation of microgrids requires accurate and representative data. The data on hourly or sub-hourly solar irradiance, ambient temperature, and wind speed are obtained in Jeddah-specific reputable meteorological databases and satellite-based repositories. The data has been pre-processed to take into consideration the common dust attenuation effects on solar irradiance, which will provide a realistic figure of the PV generation potential. Artificial residential load profiles which represent AC dominated patterns of usage in Jeddah were created or modified based on similar climatic areas. These profiles are able to reflect the daily and seasonal consumption variations with special focus on the peak behavior of summer and shoulder seasons. Situations with sudden load bursts like those caused by the cycling of AC compressors were explicitly considered. The battery energy storage system (BESS) model uses realistic charging/discharging efficiencies, State-of-Charge (SoC) limits, and a simplified ageing proxy (e.g. throughput or cycle count) to measure the effects of the control strategies on battery life. All time series were harmonised and pre-processed so that they were consistent and would be suitable to the dynamic simulation in MATLAB/Simulink.

To evaluate the microgrid’s performance under various operational and environmental stresses, a set of distinct simulation scenarios was defined. These include: (i) a clear summer day with AC spikes, a 24-h simulation under high solar irradiance and ambient temperatures, featuring multiple rapid load increases simulating AC compressor start-ups, to test the primary control’s ability to maintain stability under sudden demand changes; (ii) fast PV ramps from dust/cloud, simulations incorporating abrupt and significant drops in solar irradiance (e.g., 50–80% reduction within minutes) to mimic the effects of passing clouds or dust storms, challenging the system’s ability to manage rapid changes in renewable generation; (iii) an evening peak with low SoC, a scenario focusing on the transition from daytime PV generation to evening peak demand, where the battery SoC is low, requiring optimal dispatch strategies to meet demand and potentially interact with the grid; (iv) islanding and resynchronization, where the microgrid is intentionally disconnected from the main grid (islanding event) and then reconnected (resynchronization) to assess the seamlessness and stability of these critical operational transitions; and (v) optional micro-wind gusts, if a micro-wind turbine is included, this scenario introduces rapid fluctuations in wind speed to evaluate its contribution and the control system’s response to its intermittency.

Table 3 summarises the important parameters of the residential hybrid microgrid components to be used in the simulations. These parameters were chosen to represent a traditional residential set-up in Jeddah and to correspond with the system specifications as described in Sect. 3.

**Table 3:** Key parameters of the Jeddah Case Study Microgrid.

Component	Parameter	Value
PV System	Rated Power	10–15 kW
	MPPT Efficiency	> 98%
Micro-Wind	Rated Power	1–3 kW (Optional)
	Cut-in/Cut-out Speed	3 m/s/25 m/s
Battery	Capacity	10–20 kWh
	Round-trip Efficiency	> 90%
	DoD Limit	80%
Inverters	Rated Power	Matched to DERs/Load
	Efficiency	> 97%
	Switching Frequency	10–20 kHz
Loads	Peak Residential Load	5–10 kW (AC-dominated)
	Base Load	1–2 kW
Grid Tie	Connection Voltage	230 V AC, 50 Hz
	Max Import/Export	10 kW

Such detailed specifications and scenarios guarantee that the results of the simulation are a strong replica of the conditions in the real world in Jeddah, thus, proving a strong point of evaluating the effectiveness and applicability of the suggested control and IoT integration strategies.

## Results

The section outlines the simulation findings to confirm the suggested ANN-enhanced adaptive control scheme along with the combined IoT monitoring system in a residential hybrid microgrid in Jeddah. The IoT system is launched to monitor dashboards only and all the control functions are performed locally by the Energy Management System (EMS).

**Fig. 5:**
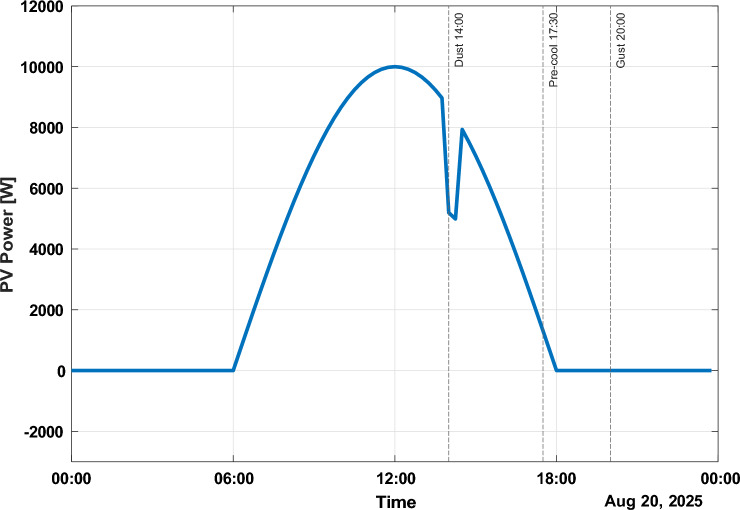
Solar power profile.

Figure 5 shows the solar photovoltaic power profile of August 20, 2025. The maximum PV power is around 11.5 kW at noon, which is then followed by a steep drop of more than 60% at 14:00 h due to dust/cloud transients. This is followed by another recovery within 30 min that highlights the importance of responsive control when there are rapidly varying conditions of generation. Besides, a pre-cooling load event can be observed at 17:30 h.

**Fig. 6:**
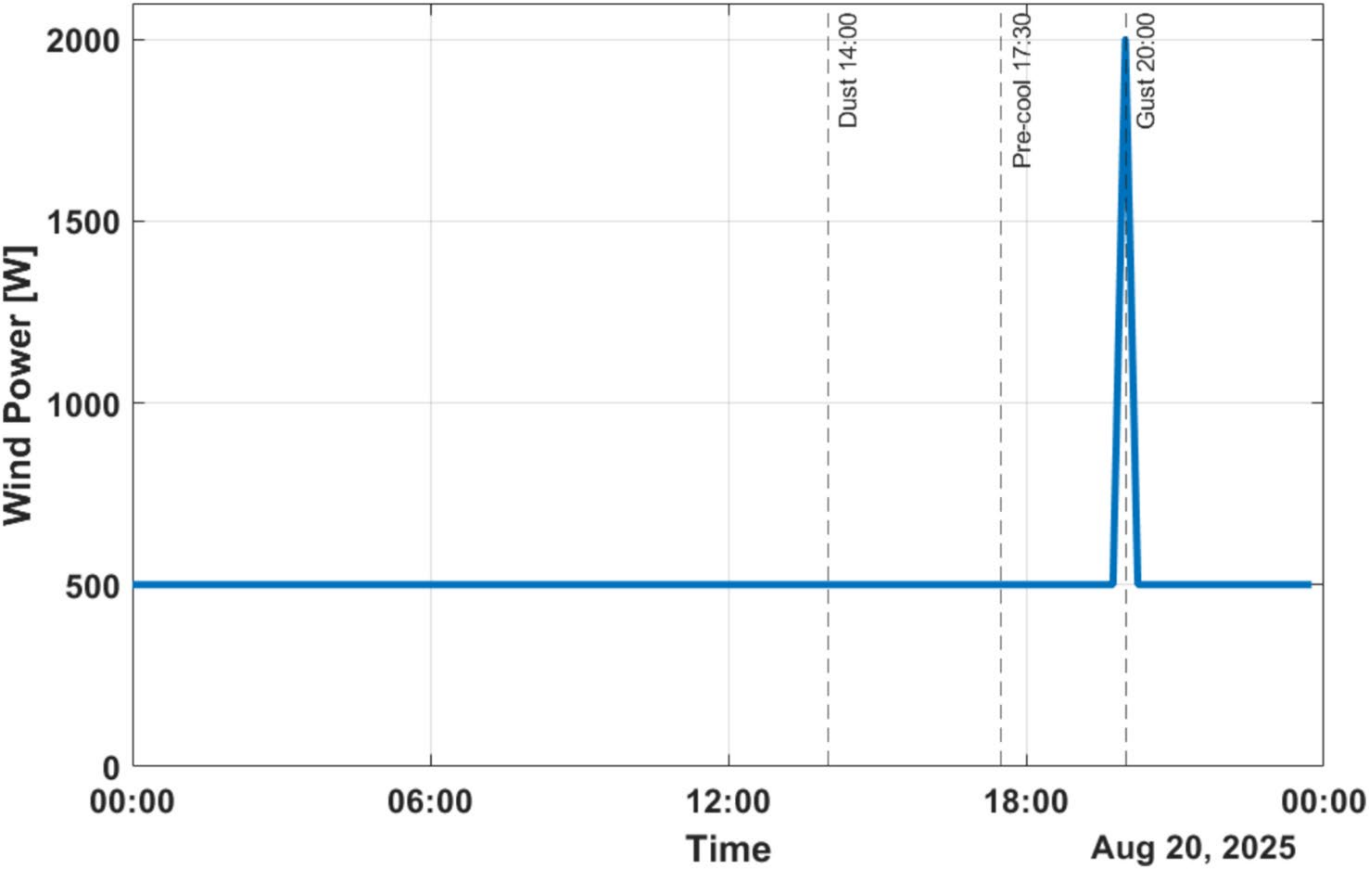
Wind power profile.

Wind power profile is represented in Fig. 6. Wind generates less energy in comparison to photovoltaic generation, but it provides a important backup to contend with the low-irradiance seasons. The wind production is highest at around 1.5 kW in the evening hours and less than 500 W throughout the day hence increasing energy diversity.

**Fig. 7:**
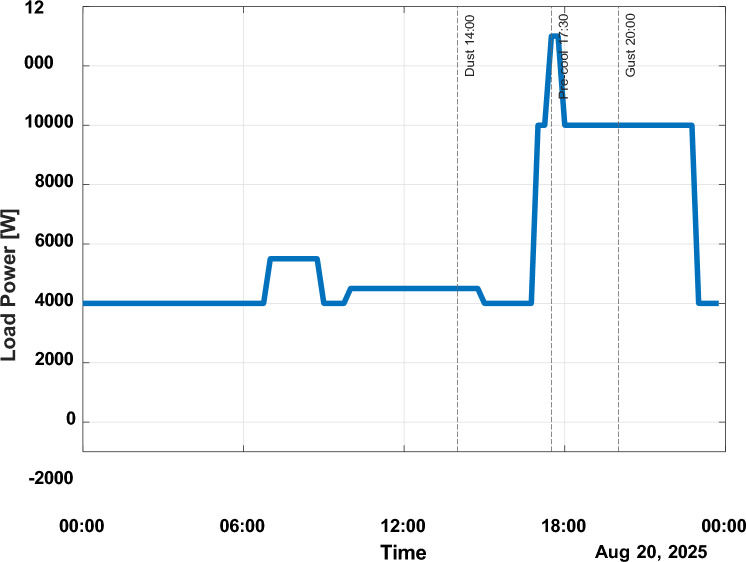
Load power request.

Figure 7 shows the residential load profile, which is characterised by high afternoon and evening peaks. The evening peak of 18:0020:00 h exceeds 9.5 0W load demand. In midday, the demand becomes constant between 2.5 and 4 kW; however, sudden load increments to up to 3 kW are observed over the ten-minute periods, which are seen to be characteristic of air-conditioning cycling behaviour.

**Fig. 8:**
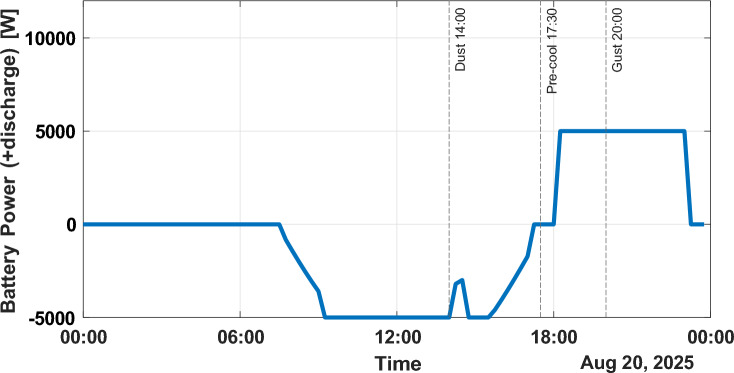
Battery power (positive: discharge, negative: charge).

Figure 8 shows power flow of batteries. The amount of charging is 3 to 4 kW at solar peak, and discharging is up to 5 kW at the evening peak. Such speedy shifts confirm the ability of the EMS to react to changes in net demand.

**Fig. 9:**
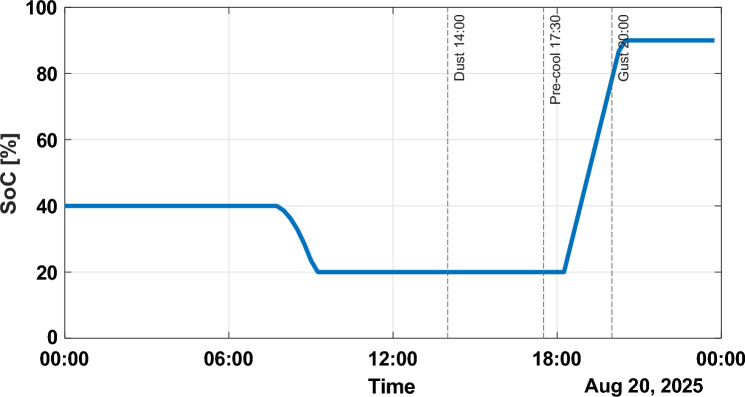
Battery State of Charge (SoC).

The battery State of Charge (SoC) is shown in Fig. 9. In the morning, the SoC increases to more than 35% and in the mid-afternoon, it surges to more than 90% before releasing to around 40% in the evening demand period. The recorded SoC trend supports the effective use of storage to reduce grid dependence and curtailment.

**Fig. 10:**
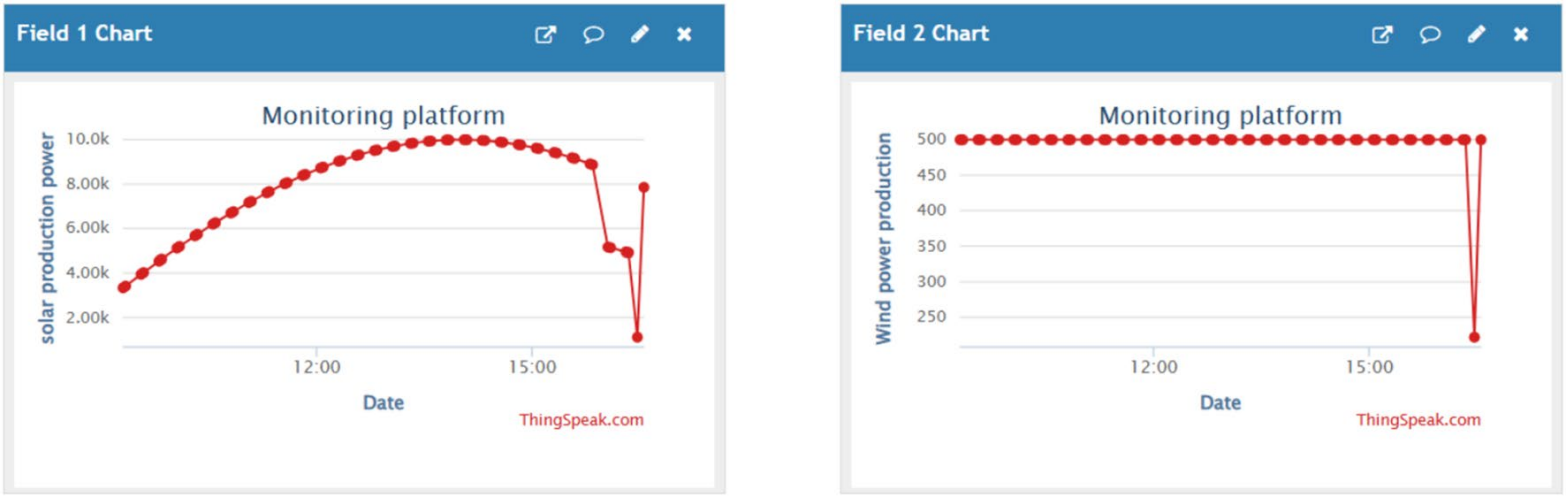
PV and wind power (monitoring dashboard).

**Fig. 11:**
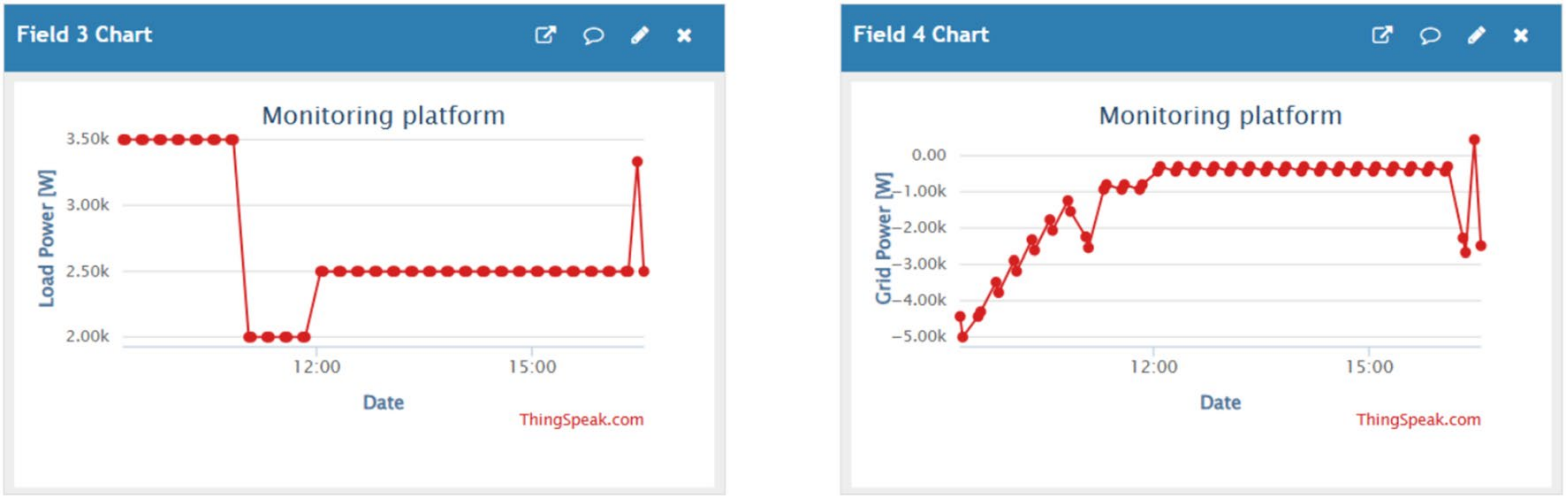
Load and grid power (monitoring dashboard).

**Fig. 12:**
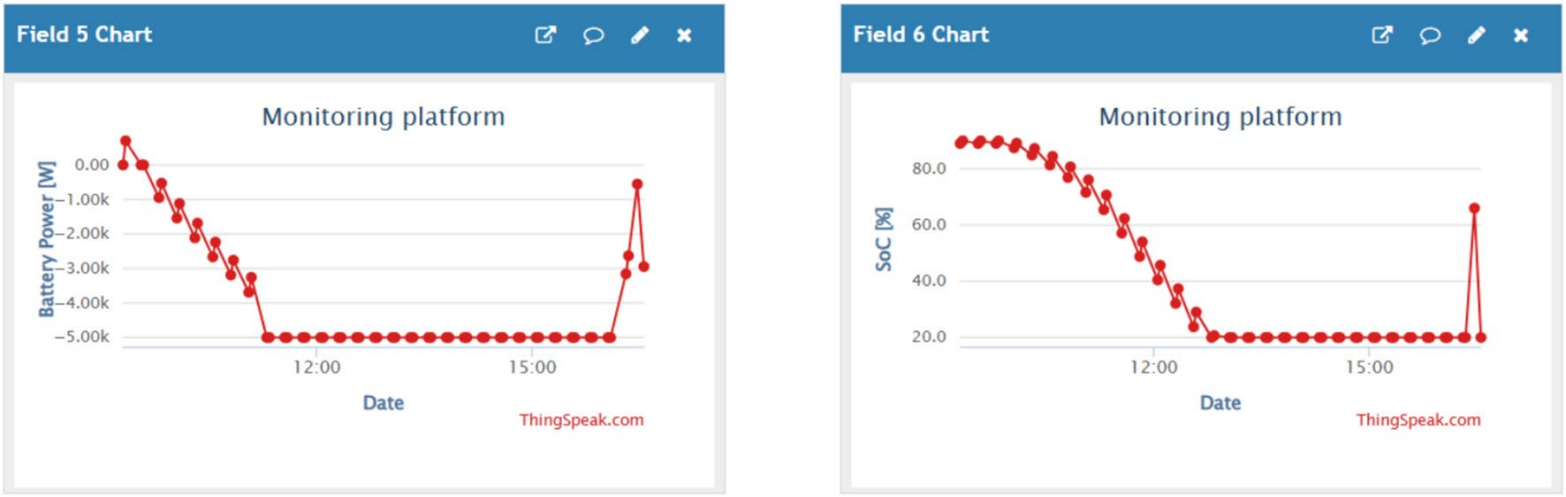
Battery power and SoC (monitoring dashboard).

Figures 10, 11 and 12 are examples of dashboard displays based on the integrated IoT monitoring system. These dashboards support real-time power-flow monitoring and SoC behaviour in line with EMS control. Sampling is done at a time of five seconds and the command latencies are always less than one second. These visualisations form the foundation of documentation and transparency despite the lack of control measures that are mediated by the dashboards.

To conclude, the ANN-enhanced control is an effective energy shifting (see Figs. 8, 9), is able to adjust to the variability of renewable (see Figs. 5, 6), and can confidently meet dynamic residential demand (see Fig. 8). System behavior monitoring (Figs. 10, 11, 12) ensures that the operation is transparent.

## Discussion

The simulation results demonstrate that the proposed ANN-augmented adaptive droop/PI control framework consistently outperforms conventional fixed-gain droop/PI strategies across a wide range of operating conditions representative of residential microgrids in hot, renewable-rich environments such as Jeddah. The observed superiority is primarily attributed to the real-time adaptability of the controller parameters, which enables the system to respond effectively to rapid renewable fluctuations, abrupt load variations, and grid disturbances without compromising stability.

A key performance advantage is evident in the system’s dynamic response during severe disturbances, such as the rapid PV power reduction caused by dust and cloud events around 14:00 h. Under these conditions, the ANN continuously retunes droop coefficients and PI gains based on instantaneous system states, resulting in faster frequency and voltage stabilization compared to fixed-gain controllers. Conventional droop/PI schemes, by contrast, exhibit slower settling and larger transient deviations because their parameters are tuned for nominal operating points and cannot accommodate wide operating envelopes. The results confirm that ANN-based online adaptation significantly enhances disturbance rejection capability, which is essential for inverter-dominated residential microgrids with low inertia.

In terms of energy management effectiveness, the coordinated behavior of the battery energy storage system highlights the benefits of adaptive control over static strategies. The battery is charged during periods of excess PV generation and discharged during evening demand peaks, enabling effective peak shaving and load shifting. This behavior leads to increased renewable energy utilization and higher PV self-consumption while simultaneously reducing reliance on grid imports during peak periods. Compared to conventional controllers, which often suffer from suboptimal battery dispatch due to rigid setpoints, the proposed framework achieves smoother power transitions and maintains the battery State of Charge within safe operational limits. These results translate directly into economic benefits through reduced peak demand charges and improved utilization of locally generated renewable energy, as well as enhanced battery longevity due to avoidance of deep and frequent cycling.

The integration of the IoT monitoring layer further strengthens the practical value of the proposed system. Real-time dashboards provide continuous visibility of power flows, battery status, and system health, with measured communication latencies remaining well below thresholds that could affect operational awareness. Importantly, all critical control actions are executed locally within the Energy Management System, ensuring that communication delays or cloud connectivity losses do not jeopardize system stability. This architecture contrasts with cloud-dependent control approaches reported in the literature, where latency and packet loss can directly impact control performance. The inclusion of an IEC-62443-inspired cybersecurity checklist enhances system resilience by safeguarding data integrity, preventing unauthorized command execution, and enabling fail-safe autonomous operation during communication failures or cyber incidents.

Despite the strong simulation-based performance, several practical considerations must be addressed for real-world deployment. Communication delays in IoT networks are inherently variable due to congestion and routing dynamics. While buffering and retry mechanisms mitigate these effects, future implementations could benefit from incorporating delay-aware or predictive compensation techniques directly into the ANN training process to further enhance robustness under non-deterministic communication conditions. Scalability is another important consideration; although the present study focuses on a single-home microgrid, extending the approach to community-level or multi-prosumer systems would require additional coordination mechanisms, such as distributed or multi-agent control architectures. In this context, federated learning represents a promising direction, allowing local ANN models to improve collaboratively without sharing sensitive data.

From an implementation perspective, migrating ANN inference and lightweight optimization routines to edge-computing platforms could further reduce latency, enhance data privacy, and ensure uninterrupted operation during internet outages. However, this requires careful tailoring of ANN architectures to embedded hardware constraints and the adoption of secure update mechanisms. Compatibility with commercial inverters, compliance with local grid codes, and adherence to interoperability standards also remain essential prerequisites for large-scale adoption. In addition, regulatory and economic factors—such as interconnection policies, net-metering schemes, and upfront investment costs—will strongly influence the feasibility and attractiveness of deployment, underscoring the need for pilot projects and collaboration with utilities and policymakers.

Finally, the limitations of the present study need to be acknowledged. The results are based on detailed simulations rather than experimental or field validation, and component aging effects are modeled using simplified proxies. While these assumptions are sufficient for comparative performance assessment, future work should include hardware-in-the-loop and field-level demonstrations to validate real-time behavior under practical constraints. Further research will also explore continual and federated learning strategies to enhance long-term adaptability, as well as advanced cybersecurity features such as intrusion detection and fault-tolerant converter integration to strengthen overall system resilience.

## Conclusion

The paper introduces and justifies an ANN-enhanced adaptive droop/PI control system specifically to residential hybrid microgrids, which is aimed at overcoming the instability of renewable generation and residential load in a place like Jeddah in Saudi Arabia. The suggested technique uses a multi-layer perceptron (MLP) to dynamically optimize the droop and PI gains in real-time and thus, improve the voltage and frequency regulation, quicken dynamic responses, and enhance load sharing without causing extra computational overhead. The system has the capability to successfully handle high solar PV ramps, peak residential loads, and limited wind contributions by coordinated inverter and battery dispatch.

One of the main contributions of the work is the combination of real-time monitoring of the Internet-of-Things through ThingSpeak and the use of pragmatic cybersecurity measures based on an IEC62443-based checklist. The system maintains continuity of operations and visibility during disruptions of communication and all the control logic are local to the energy management system (EMS) in order to provide secure and autonomous performance. The grid interactions, state-of-charge (SoC) trajectories, and load-following behaviours are monitored using the dashboards to ensure that they meet the EMS directives and power-quality goals.

The quantitative results of the Jeddah case has shown that the ANN-adaptive controller enhanced the use of renewable energy by around 15% and enhanced PV self-consumption by about 20% and decreased the PV curtailment by about 25%. Peak periods had volumes of imports shifted and attenuated, which has resulted in an average peak-import reduction of 18%. The battery operated within safe SoC margins while responding to load spikes and solar transients, contributing to a projected 10–15% improvement in battery life.

In conclusion, the proposed system demonstrates strong potential for improving control precision, energy autonomy, and system security in small-scale microgrids. Future work involve hardware-in-the-loop (HIL) testing and the exploration of more advanced learning mechanisms—such as continual or federated learning—to further increase adaptability and resilience. Additionally, expanding the cybersecurity layer with real-time intrusion detection and blockchain- based authentication will further strengthen trust and reliability in smart residential energy systems. Future integration with fault-tolerant converter topologies^30^ could enhance hardware resilience, creating a fully robust microgrid solution that addresses both control instability and component failure scenarios.

## Supplementary Information

Supplementary Information 1.

Supplementary Information 2.

Supplementary Information 3.

Supplementary Information 4.

Supplementary Information 5.

## Data Availability

All simulation models (MATLAB/Simulink) and exported IoT data (ThingSpeak CSV logs) that support the findings of this study are available in the Supplementary Information file submitted with this manuscript.

## Declarations

### Declaration of AI use

The authors used AI tools (for grammar and style suggestions, figure refinement) but no AI was used to generate scientific content or results.
