# Supplementary figures and images for: ANN-augmented adaptive droop/PI control for residential hybrid microgrids with IoT monitoring
Source: Sci Rep. 2026 Apr 10;16:16960. doi: 10.1038/s41598-026-46557-z (PMC13230602; doi:10.1038/s41598-026-46557-z)

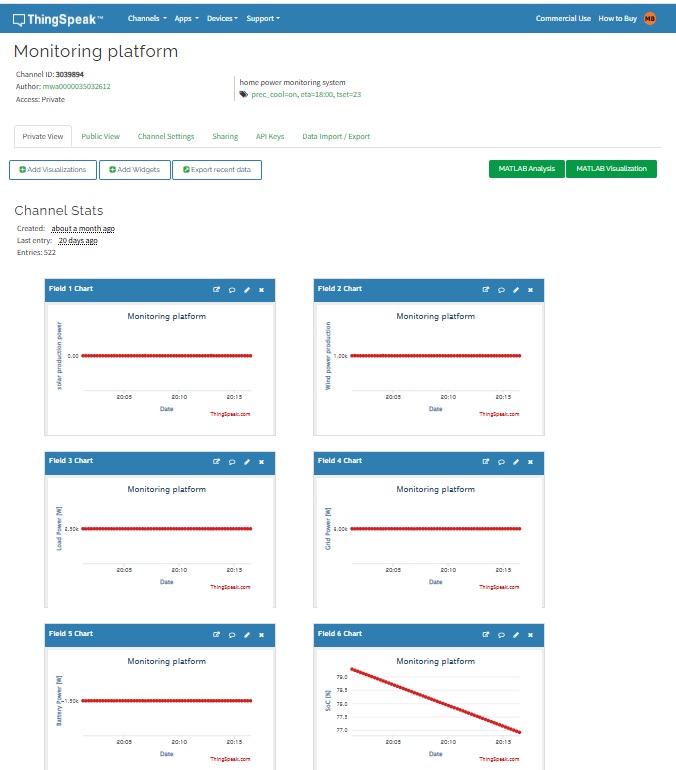

Supplement: Supplementary file 2 — Supplementary Information 2. [file 41598_2026_46557_MOESM2_ESM.jpg]

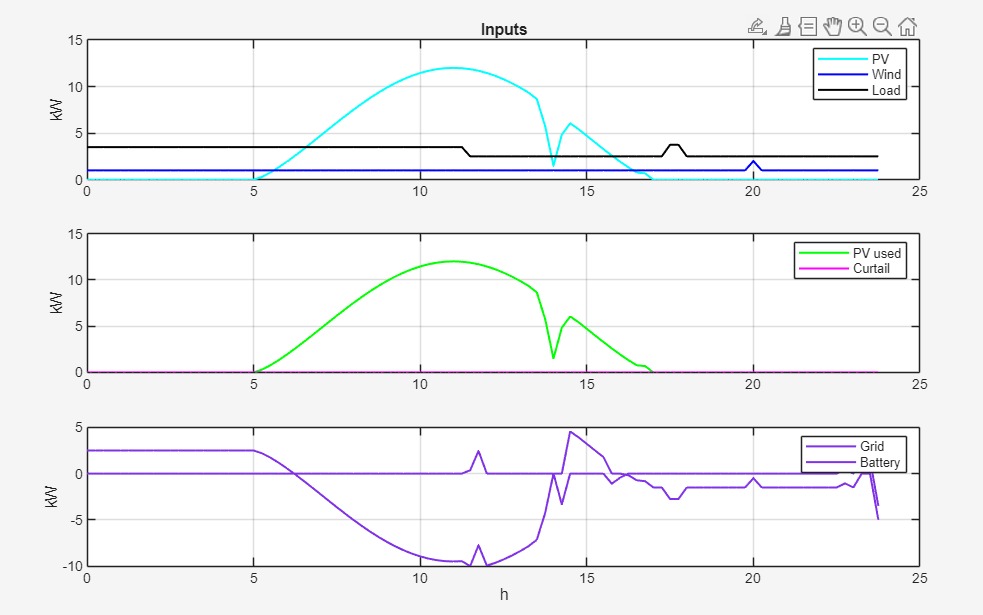

Supplement: Supplementary file 4 — Supplementary Information 4. [file 41598_2026_46557_MOESM4_ESM.jpeg]

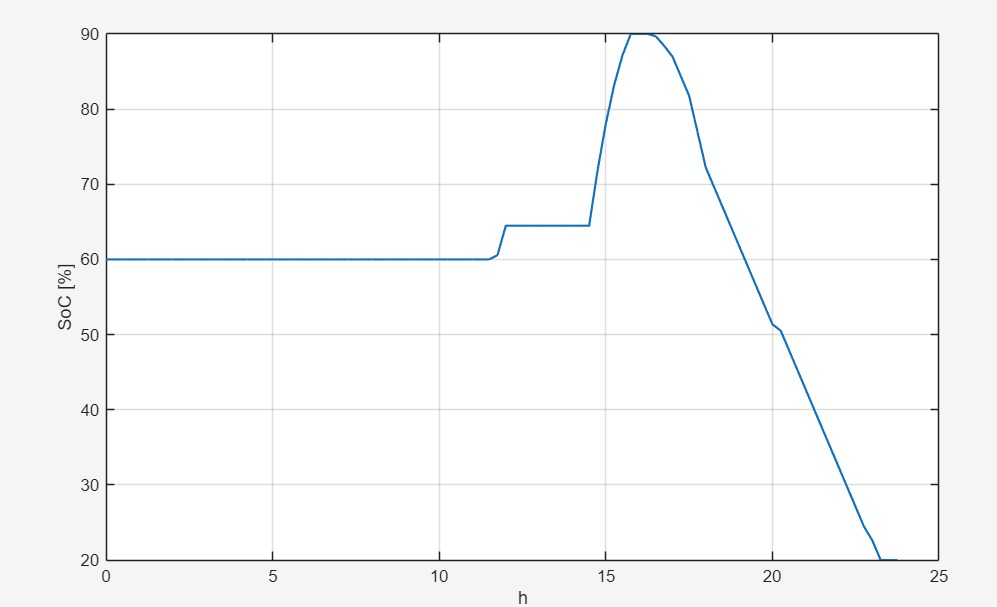

Supplement: Supplementary file 5 — Supplementary Information 5. [file 41598_2026_46557_MOESM5_ESM.jpeg]
